# Physical features of Adam’s Bridge interpreted from ICESat-2 based high-resolution digital bathymetric elevation model

**DOI:** 10.1038/s41598-024-65908-2

**Published:** 2024-06-28

**Authors:** Giribabu Dandabathula, Koushik Ghosh, Rohit Hari, Jayant Sharma, Aryan Sharma, Niyati Padiyar, Anisha Poonia, Apurba Kumar Bera, Sushil Kumar Srivastav, Prakash Chauhan

**Affiliations:** 1 Regional Remote Sensing Centre - West, National Remote Sensing Centre, Indian Space Research Organisation, Jodhpur, India; 2 Computer Science Department, Jaipur Engineering College and Research Centre (JECRC) University, Jaipur, India; 3 Department of Geography, Panjab University, Chandigarh, India; 4 Amity Institute of Geoinformatics and Remote Sensing, Amity University, Noida, India; 5 Regional Centres, National Remote Sensing Centre, Indian Space Research Organisation, New Delhi, India; 6 National Remote Sensing Centre, Indian Space Research Organisation, Hyderabad, India

**Keywords:** Limnology, Ocean sciences, Solid Earth sciences

## Abstract

Adam’s Bridge is a submerged ridge connecting India and Sri Lanka, generally regarded as a chain of shoals extending for ~ 29 km from Dhanushkodi on the Indian side to Talaimannar Island of Sri Lanka. A high-resolution digital bathymetric elevation model generated using the seafloor returned photons of ICESat-2 was used to understand the intricate details of Adam’s Bridge structure. Photons emanating from ICESat-2’s green laser have the potential to detect the seafloor up to a depth of ~ 40 m; taking a cue from this potentiality, in our research, we have accrued ~ 0.2 million photons representing the depth information and generated a 10 m resolution bathymetric data for the extent of Adam’s Bridge. Visual interpretations made from this bathymetric data through 3D perspectives with multi-directional lighting effects, and also with the derived parameters like contours, slope, and volumetric analysis, enabled us to recognize the current form of Adam’s Bridge’s physical features. The results from our research confirm that, in its entirety, Adam’s Bridge is a submarine continuation of Dhanushkodi and Talaimannar Island. Throughout the crest line of Adam’s Bridge, approximately 1.5 km on either side is highly undulating within the super-shallow water with occurrences of sudden depths. There is an asymmetry of transverse slopes to the base on both sides of Adam’s Bridge, indicating dominant transgression of material energy from the waters of the Gulf of Mannar compared to the Palk Strait. The volume of Adam’s Bridge computed in our research yielded a value of ~ 1 km^3^; interestingly, only 0.02 percent of this volume is above the mean sea level, and in general, the same is visible in optical satellite imagery—in total ~ 99.98 percent of the Adam’s Bridge is submerged in shallow and super-shallow waters.

## Introduction

Adam’s Bridge spans from Dhanushkodi, a south-eastern point of Rameshwaram Island in India, to the north-western end of Talaimannar in Mannar Island of Sri Lanka, is an isthmus mostly submerged in the shallow waters (depths less than 10 m) of the epipelagic zone of the Indian Ocean. Extending up to a length of ~ 29 km, Adam’s Bridge is constituted with a remarkable chain of loose and fine sandbanks exposed above the water level at irregular intervals of its length. These sandbanks generally exhibit seasonal shifts; importantly, none have the slightest presence of rocks and vegetation^1–6^.

The north of the Adam’s Bridge extends Palk Bay, which is continuity to the Palk Strait—an inlet of the Bay of Bengal. Palk Strait is a shallow water body with an average depth ranging from 9 to 12 m; however, random sinks up to 14 m depth exist throughout^7,8^. Palk Bay is bounded east by the Sri Lankan coast and south by Mannar Island, Adam’s Bridge, and Pamban Island^7,8^. The southern part of Adam’s Bridge has the Gulf of Mannar, an arm of the Indian Ocean, which is a Marine Biosphere Reserve under the Man and Biosphere program of the United Nations Educational, Scientific and Cultural Organization (UNESCO) and is considered one of the world’s richest regions from a marine biodiversity perspective^9–12^. The seafloor’s depth for this gulf extends up to 2280 m from the coast with regular outline^13–15^. The water currents of both the Gulf of Mannar and Palk Strait are characterized by bi-annually reversing monsoon winds (south-west monsoon from early June and north-east monsoon post-October) resulting from seasonal differential heating and cooling of the continental land mass and the ocean^16,17^. In the context of Adam’s Bridge, the currents due to the prevalent winds from annually reversing monsoon winds will have a significant bearing on its water levels and drifting surface sands; ultimately, it is the reason for sand banks’ shifting phenomenon that occurs periodically.

During the nineteenth century, British surveyors did extensive fieldwork along the Adam’s Bridge. Drilling bores for geological investigation is also a part of these surveys. These surveys resulted in maps used for input to various committees to discuss the feasibility of dredging this causeway. The plans for the dredging arose to facilitate large draught ships to navigate across the Gulf of Mannar and Palk Strait, thus reducing the distance of navigation by avoiding circumnavigation around the Sri Lankan waters^18–20^. Surveys are also done to plan a railway line connecting Dhanushkodi and Mannar Island^1,5,21^. However, none of these proposed activities have success in implementation, but the borings done as a part of their surveys have yielded information regarding the material strata of Adam’s Bridge at a few places along its length. From the field survey and borings, surveyors have observed sand up to a depth of 7–9 m. Further to this depth, it either reached the base containing the Holocene conglomerate or calcareous sandstone (chiefly by the agency of Polyzoa and Nullipores)^4^. At some drillings, the base is accompanied by coral reefs in all stages of decay, from the living forms to almost structureless limestones^1,4,5^. Certain borings have found traces of third strata containing a tessellated platform of the coralline sand layer before reaching the continued compact formation^4,22,23^.

From the ages, with reference to the Hindu mythology called Ramayana, in most South Asian countries, this geographic extent is referred to as Ram Setu^24^. Persian navigators referred to this extent as ‘Setu Bandhai,’ or bridge on the sea connecting India and Sri Lanka—a prevailing name used by the people of the local states attributing the confluence of the Gulf of Mannar and Palk Strait^24^. Major James Rennel (1742–1830), the Surveyor General of Bengal for East India Company, labeled this geographic extent as Adam’s Bridge in all the provincial maps; however, by that time, many European navigators were already referring to this extent by the same name^21^.

**Figure 1:**
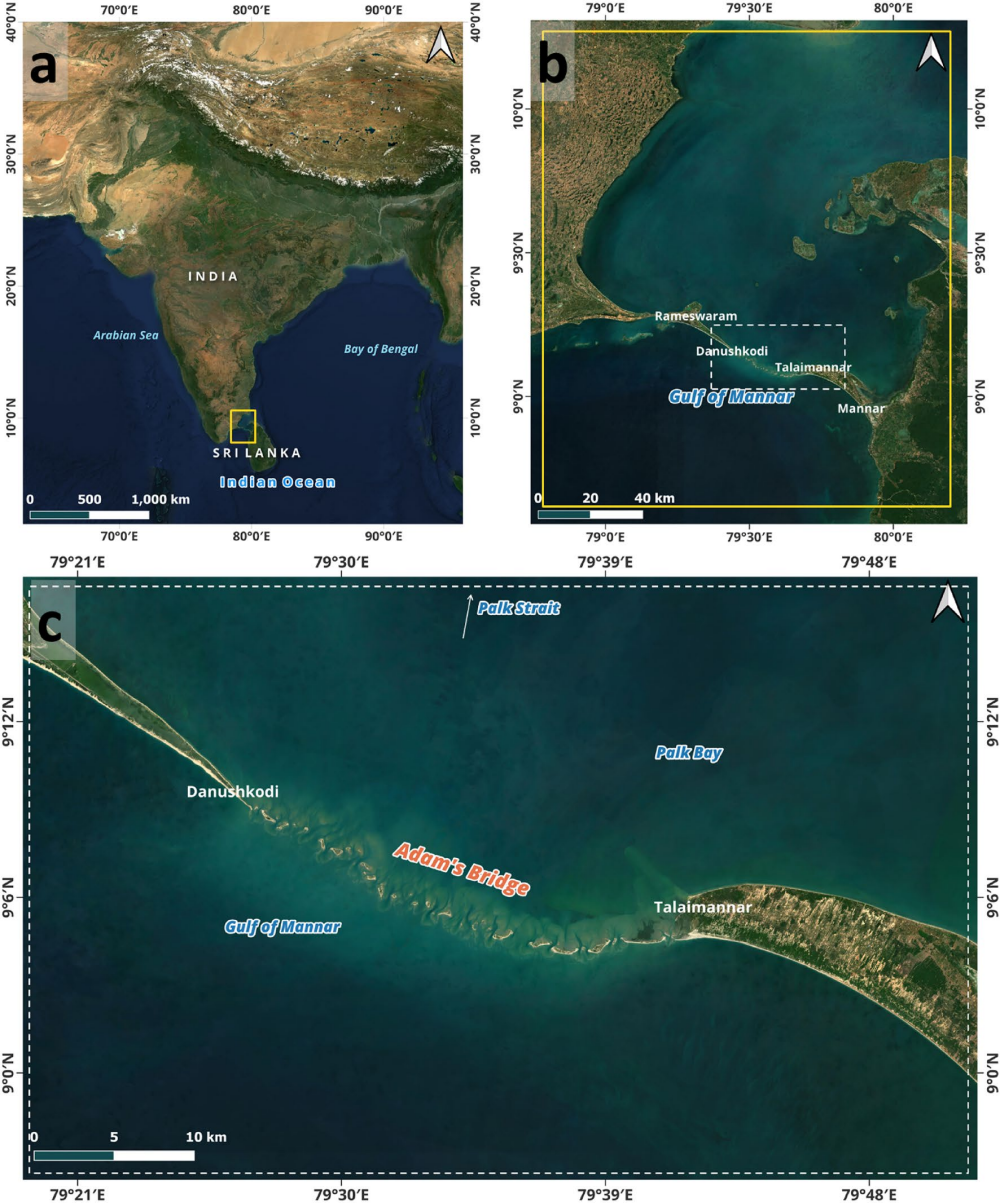
Location map of Adam’s Bridge. (**a**) The map shows India and Sri Lanka by highlighting the extent of Adam’s Bridge with a yellow box. (**b**) The map shows Rameshwaram Island (Indian side) and Mannar Island (Sri Lankan side) and highlights Adam’s Bridge with a white box that constitutes the study area. (**c**) Satellite Image showing Adam’s Bridge, which stretches from Dhanushkodi to Talaimannar. *Source* Map prepared using the satellite imagery through web mapping services (https://tiles.maps.eox.at/wms)* of the Sentinel-2 cloudless layer for 2021 by EOX ( and ). Maps were composed using QGIS Ver. 3.30.0-'s-Hertogenbosch (). *This work is licensed under a Creative Commons by Attribution (CC BY-NC-SA 4.0) license.

Figure 1 shows the location map of Adam’s Bridge in high-resolution satellite imagery acquired by the Sentinel-2 mission, developed and operated by the European Space Agency. Optical remote sensing data use visible, near-infrared, and short-wave infrared sensors to provide information about Earth’s surface features but have limitations in providing details about submerged features. Earlier researchers have interpreted and reported the details about exposed sandbanks of Adam’s Bridge using optical remote sensing data^25–27^. However, to understand the intricate structural details of Adam’s Bridge sort of reefal assemblage – primarily, a submerged feature, there is a need for high-resolution bathymetric data^16,28^. Open-access global bathymetric data sources like the General Bathymetric Chart of the Oceans (GEBCO) (https://www.gebco.net/) and Global Multi-Resolution Topography (GMRT) (https://www.gmrt.org/) are available at ~ 450 m and 100 m respectively. Dandabathula et al.^29^, while evaluating the performance of the latest GEBCO bathymetric dataset at the extent of Adam’s Bridge and its surroundings, have highlighted that the seafloor retrieved from the GEBCO_2023 grid exhibits erratic fluctuations within the water column when compared with a reference dataset. On the other hand, GMRT datasets are synthesized solely using multibeam sonar data collected by scientists and institutions worldwide, which is reviewed, processed, and gridded by the GMRT team and merged into a single continuous updated compilation of global elevation data (https://www.gmrt.org/); due to the lack of multibeam sonar data by any institution, at the vicinity of Adam’s Bridge and surroundings the complied GMRT bathymetric data consists of only hypothetical values in the said extent. Thus, the GEBCO and GMRT, due to their coarser resolution and seafloor of speculated nature, may not help depict and understand the intricate details of Adam’s Bridge. There is a need to generate a bathymetric dataset for the extent of Adam’s Bridge, which can help to portray the seafloor to understand its physical structure.

Technologies that can generate bathymetric data include ship-borne sounding sensors, satellite-derived empirical methods, and aerial/space-borne altimeters^30–34^. In recent times, there has been thrust in advocacy to use Unmanned Surface Vehicles (USVs) and Autonomous Underwater Vehicles (AUVs) for mapping the seafloor; however, these technologies are still in the early stage of adoption, besides having cost implications^33,34^. While each of these techniques has its own merits and demerits, ship-borne seabed topography mapping remained a widely used method, though restricted its applications in shallow water areas as sonar mounting devises poses challenges during the data acquisition, and also, the acoustic signals get distorted, impacting the measurement accuracy^33,34^; because of this, for the extent of Adam’s Bridge, as it is mainly submerged in shallow waters, there is very minimal availability of ship-borne sounding data (that too by research boats) within its vicinity. To date, the lack of high-density bathymetric information on the extent of Adam’s Bridge dents the understanding of its physical structure.

Space-borne LiDAR data has several advantages in terms of high precision, low cost, and, importantly, its ability to detect depths for shallow waters^35^. However, the limitation of LiDAR data for bathymetric studies is that its efficiency capability decreases in deep-water environments and turbid load water^33^.

NASA’s Ice, Cloud, and Land Elevation Satellite-2 (ICESat-2) is a new generation space-borne laser altimeter that has been operational since September 2018 and hosts a single sensor, namely, Advanced Topographic Laser Altimeter System (ATLAS)^36,37^. ATLAS is a photon-counting LiDAR sensitive to single-photon reflections that uses three beam pairs (six beams) to obtain nearly contiguous along-track measurements for a 0.7 m sampling distance. ATLAS instrument transmits green (532 nm) laser pulses at 10 kHz from the nominal ICESat-2 ~ 500 km orbit to provide the range measurements from space^36–38^. Combined with the satellite precision pointing and positioning information, the range measurements produce geolocation and elevations for all the successful photon returns from the Earth’s surface^36^. A variety of geophysical data products that are specific to Earth’s surface features (like land, ice, sea ice, vegetation/canopy, inland surface water, and ocean surface height) are processed by the ICESat-2 science teams and disseminated through a web portal maintained by NASA National Snow and Ice Data Center Distributed Active Archive Center (NSIDC DAAC) (https://nsidc.org/data/icesat-2/products). A Level-2A data product, ATL03, contains height above the WGS84 ellipsoid with the mean sea surface, latitude, longitude, and time for all photons downlinked by the ATLAS instrument onboard ICESat-2 observatory^39^. The ATL03 product design aims to become a single source for all photon data and ancillary information that other higher-level ICESat-2 products need^39^. Importantly, as per the ICESat-2 ATL03’S Algorithm Theoretical Basis Document (ATBD), the essential time-variable geophysical parameters will undergo various corrections that nullify the effects of ocean tides, solid earth tides, ocean loading, and solid earth pole tides^39^; these corrections in the context of deriving the bathymetric information signifies the applicability of ICESat-2 photon data to perceive accurate depth information and mitigate the impact of temporal variations while considering datasets acquired on different dates.

In the first five years after the launch of ICESat-2, numerous research areas in the Earth sciences reported the significance and application capability of geolocated photon data^40^—in fact, the geolocated photon data from ICESat-2 not only filled certain gap areas in the geospatial technologies but also unleashed novel applications for Earth sciences (https://icesat-2.gsfc.nasa.gov/publications). Although the primary goal of ICESat-2 is to monitor the cryosphere, due to the photon penetrability of up to ~ 40 m in clear water, bathymetric mapping of shallow waters has become a promising application^41–51^. Various validation procedures using either in situ or reference data have reported accuracies of ICESat-2’s seafloor depth in the range of 0.2–0.8 m^41–51^; this led to operational methodologies to derive the seafloor depths from the ICESat-2 photons^29,41,52,53^.

For this research to understand the intricate details of Adam’s Bridge, a high-resolution (10 m) Digital Bathymetric Elevation Model (DBEM) was generated for its extent. The source of this DBEM is a point database that contains majorly ~ 0.2 million depth values contributed by ICESat-2 photons and sounding depths retrieved from Electronic Navigation Charts (ENCs) and hydrography charts. The study area contains islands like Rameshwaram and Talaimannar; the elevation values for these islands were considered from an open-access bare-earth model. Subsequently, this point database was interpolated to generate a bathymetric surface.

Three hundred ninety-six beams from seven ground reference tracks (https://icesat-2.gsfc.nasa.gov/science/specs#) of ICESat-2 acquired between October 2018 and July 2023 over the extent of Adam’s Bridge have qualified to abide by the prerequisite conditions needed for successful bathymetric measurements; generally, these prerequisite conditions include a preference for acquisitions during night-times and seasons of low turbid load in water^41,43,54^. ICESat-2 acquisition method yields along-track data, and solely using along-track points may not produce a continuous surface through interpolation methods; thus, additional points must compensate for the distribution. For this, depth values were retrieved from published ENCs and hydrography charts available from authorities^55–57^. For the extent of having land part, elevation values from the open-access bare-earth model called Forest And Buildings removed Copernicus DEM (FABDEM)^58,59^ have contributed to compensate for the point distribution issue. Technical validation for the seafloor depths retrieved from the ICESat-2 water-penetrated photons was done per the recommended procedures^60^ using the reference datasets at two test sites (described in the methodology section). Similarly, the final DBEM was validated using the reference data available in the form of sounding depths from the hydrographic chart generated by the Indian Naval Hydrographic Office (INHO) (https://hydrobharat.gov.in/).

The above DBEM was used to recognize and describe the physical features of Adam’s Bridge—the core objective of this article. A three-dimensional (3D) perspective view aids in the visual analysis of digital surface data and is a promising approach for ocean data exploration, analysis, and understanding of seabed topography^61^. In addition, 3D perspective views with multi-directional lighting effects enhance the impressions of relief data^62^; thus, towards perceiving the physical features of the submerged assemblage of Adam’s Bridge, multi-directional lighting effects in a 3D visualization platform available in ESRI’s ArcScene software (www.esri.com) was used for interpretation. DBEM can be a source for deriving contours, slopes, and aspects; these derived data act as quantitative parameters of the seafloor^63^. Thus, the physical features of Adam’s Bridge are reported in this article through the interpretations from DBEM and its derived parameters.

## Results and discussion

**Figure 2:**
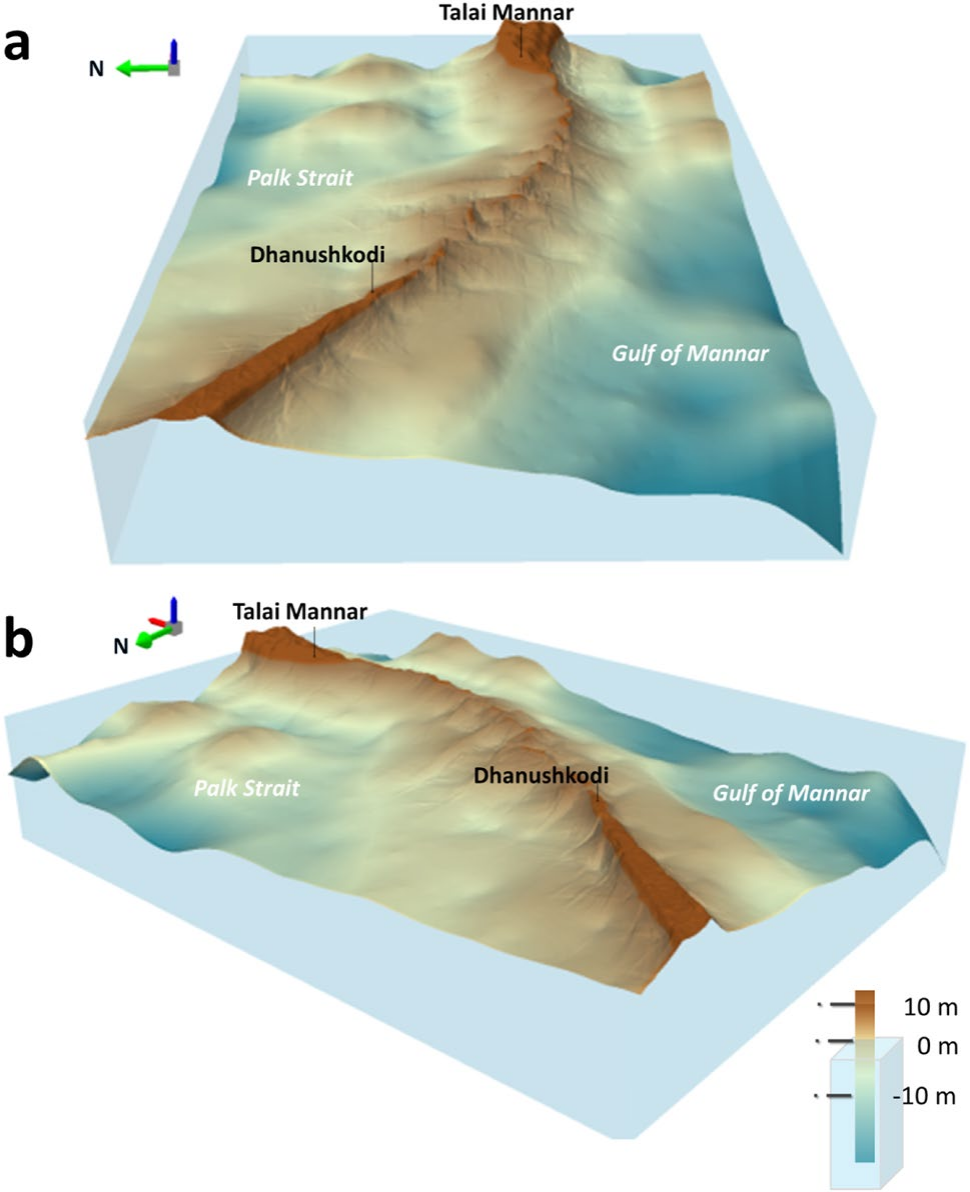
Three-dimensional perspective views of digital bathymetric elevation model generated using ICESat-2 water-penetrated photons for Adam’s Bridge. (**a**) Perspective view of Adam’s Bridge from the Gulf of Mannar as an observer position. (**b**) Perspective view of Adam’s Bridge from Palk Strait as an observer position. The primary inference from both these perspectives is that in its entire form, Adam’s Bridge is a submerged ridge with a submarine continuation of Dhanushkodi and Talaimannar Island. The exposed sand banks are only 0.02 percent of Adam’s Bridge’s total volume when the base is considered at 8 m depth. These perspectives were generated using ESRI’s ArcScene Ver. 10.8.1 software (https://www.esri.com/).

**Figure 3:**
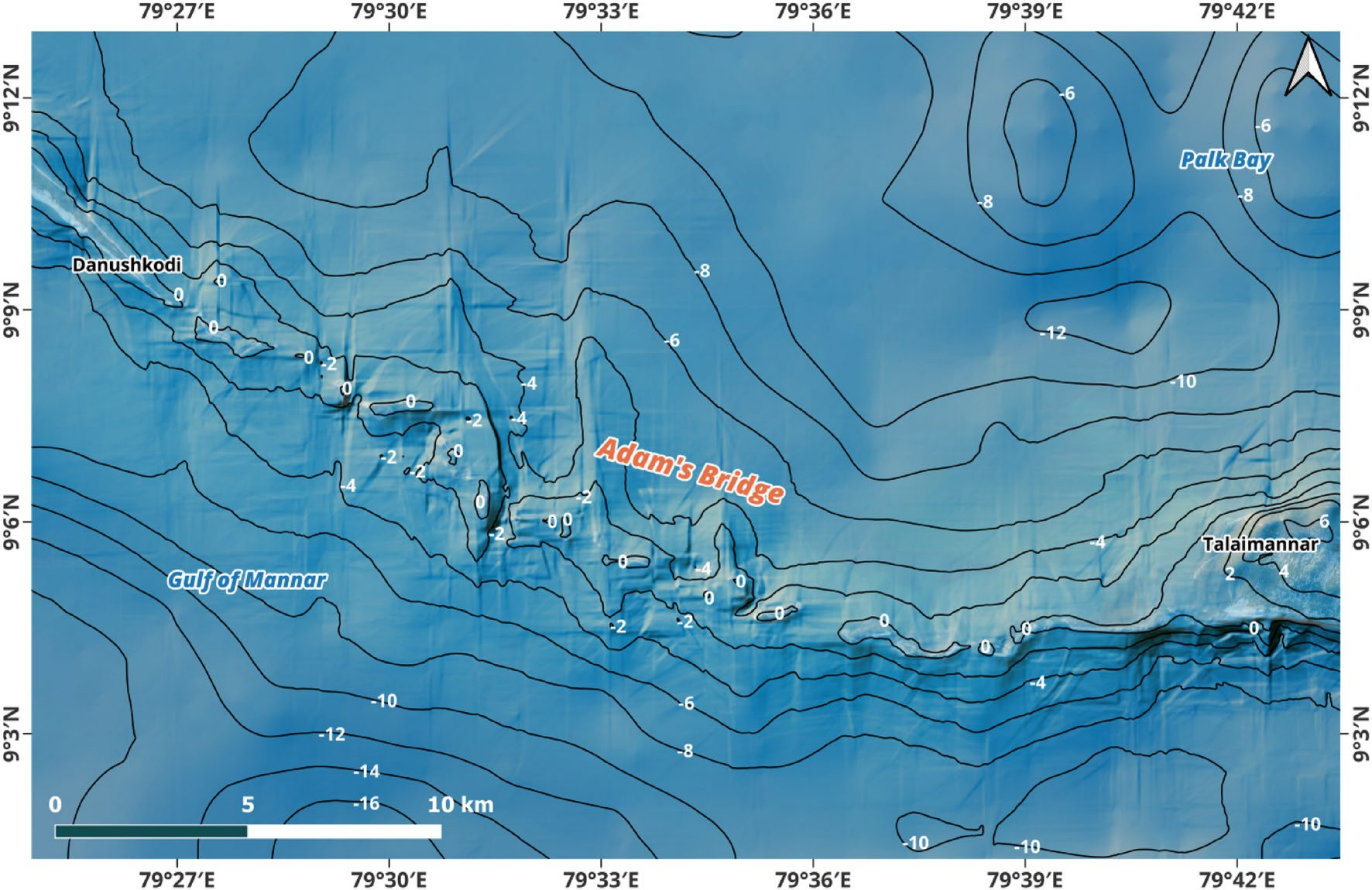
Contour map of 2 m interval for the extent of Adam’s Bridge derived from the digital bathymetric elevation model generated using ICESat-2 water-penetrated photons. The topographic surface on either side for 1.5 km of the Adam’s Bridge’s crest line is highly undulating with sudden depths. Towards the Gulf of Mannar side, from Dhanushkodi’s tip, the distance from the crest of Adam’s Bridge to the − 8 m contour line is 6.5 km for its initial 17 km. Towards reaching the Talaimannar side, the distance to the base fluctuates between 2.5 and 3.5 km. Towards the Palk Strait side, the distance from the crest to the base is always greater than 7 km.

**Figure 4:**
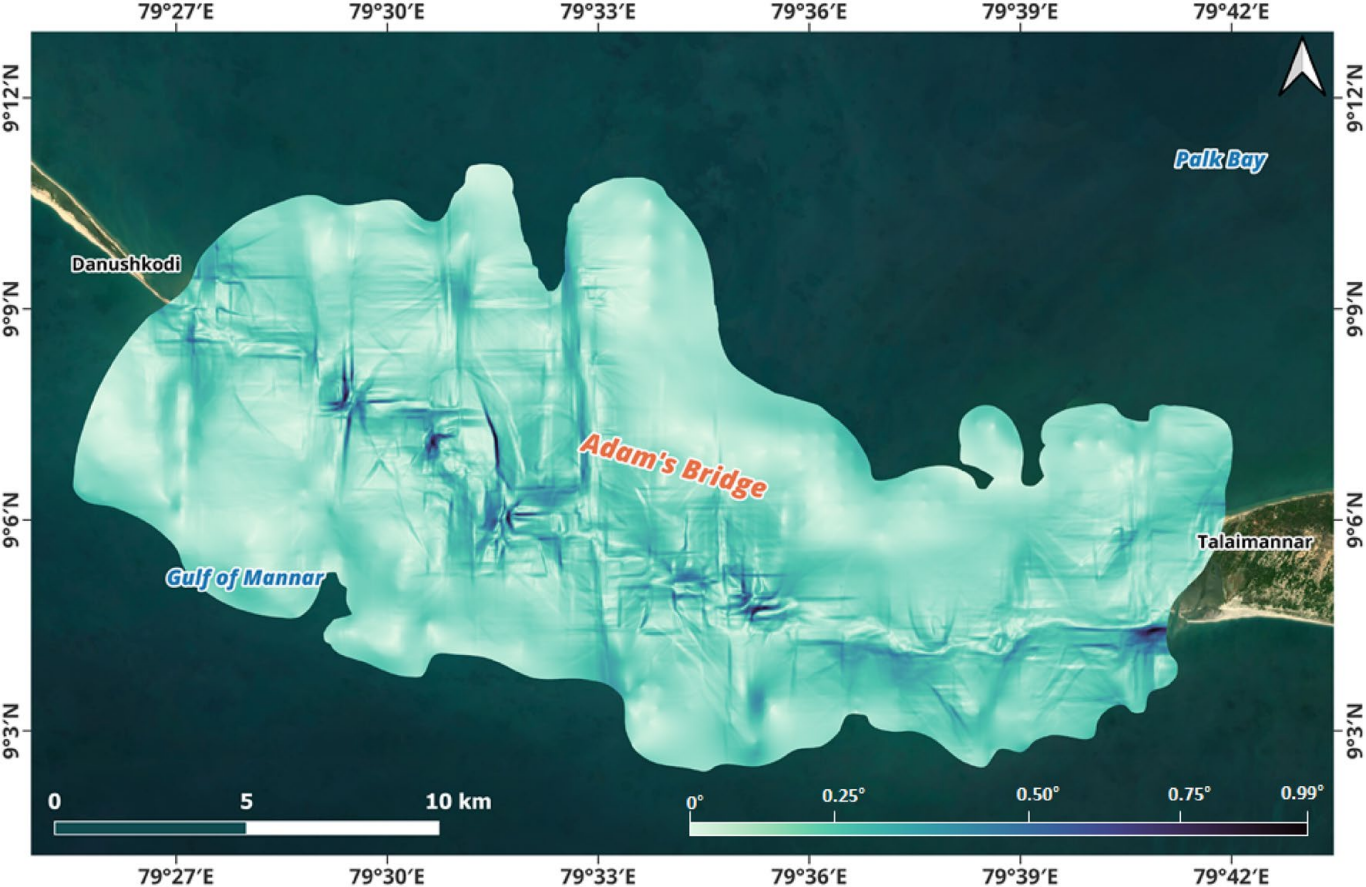
Slope map for the extent of Adam’s Bridge derived from the digital bathymetric elevation model generated using ICESat-2 water-penetrated photons. The transverse slope towards the Gulf of Mannar mainly varies between 0.05° and 0.35°. In contrast, towards the side of Palk Strait, the slope, on average, is never greater than ~ 0.2°.

Figure 2 shows 3D perspective views of the DBEM generated for the extent of Adam’s Bridge. Specifically, Fig. 2a and b are two different perspectives of Adam’s Bridge, one with the observer position from the Gulf of Mannar and the other from Palk Strait. Figures 3 and 4 show a contour map of a 2 m interval and a slope map derived from the DBEM of the study area, respectively.

One important observation from the interpretation of the DBEM is that, in its entire form, Adam’s Bridge is a submerged ridge with a submarine continuation of Dhanushkodi and Talaimannar Island (refer to Figs. 2 and 3) from a rationalized depth of 8 m. From the tip of Dhanushkodi, Adam’s Bridge’s general trending long axis is oriented from west-northwest to east-southeast in secondary intercardinal directions, entirely perpendicular to the predominant wave approach directions of both the Gulf of Mannar and the Palk Strait. The general direction of Adam’s Bridge for the first 17 km of its length is about east-south-east; later, the direction is gradually curved towards the north of the east and finally touches Talaimannar Island at its east.

Along the Adam’s Bridge crest, which occupies a central position from its base on either side reckoning from sea level to 8 m (treated as a base), the transverse slope towards the Gulf of Mannar mainly varies between 0.05° and 0.25°—implying that for every 10 m horizontal distance, there is a change in slope; however, the slope towards Talaimannar side is varying between 0.2° and 0.35°. In contrast, towards the side of Palk Strait, the slope, on average, is never greater than ~ 0.2° (refer to Fig. 4). The topographic surface on either side of the Adam’s Bridge’s crest line for an approximate length of 1.5 km is highly undulating with varying slopes and sudden depths (refer to Figs. 3 and 4). Towards the Gulf of Mannar side, from Dhanushkodi’s tip, the distance from the crest of Adam’s Bridge to the -8 m contour line is 6.5 km for its initial 17 km. For the further length, the distance to the base fluctuates from 2.5 to 3.5 km. Meanwhile, the distance from the crest to the base is always greater than 7 km towards the Palk Strait side. The rationale for treating the depth to 8 m is that from this level, the seafloor of Palk Strait exhibits flatness; however, from the rationalized base of Adam’s Bridge, the seafloor depth in the Gulf of Mannar stretches as a low platform, deepening fair and evenly to the south at about a rate of 2 m depth per 1 km to 36 m, after which it sinks more rapidly to great depths.

Geologically, the genesis of India and Sri Lanka are closely linked, from being a part of the ancient supercontinent of Gondwana and later with the ancient supercontinent of Pangea during the Permian period^64–66^. Under the force of plate tectonics during the Triassic period, Pangea separated into two major supercontinents, Laurasia and southern Gondwana, where India, in association with Sri Lanka, emerged as one of the huge land masses in the southern Gondwana^66,67^. India drifted northwards as an isolated island in the Thethys Seas, crashing into Laurasia about 35–55 million years ago to take up its present position^67^. From the Triassic to the Pleistocene and Holocene, sea levels have repeatedly submerged or emerged the land bridge connecting India and Sri Lanka^67,68^. The land bridge may result from conditions associated with the sea level fluctuations since the last deglaciation associated geologic and tectonic settings. Currently, this land bridge connectivity is in a zone of active wave attacks from both sides^16^, i.e., the Gulf of Mannar and the Palk Strait. Adam’s Bridge’s current form might be significantly influenced by the sources of sedimentation/sand and their to and fro movements, pattern of currents, and energy to move the sedimentation^69–71^ from the Gulf of Mannar and the Palk Strait and vice versa; these parameters may have resulted in maintaining the asymmetrical slopes on either side and aggregation of fair sand deposits on its crest and sides^25^. Generally, high waves are observed in the Gulf of Mannar, particularly during the southwest monsoon period compared to the Palk Strait, and occasionally, swells dominate in the Gulf of Mannar^72^.

Previously, researchers have attempted investigations to understand the wave patterns and currents that originated in the Gulf of Mannar and Palk Strait^16,17,27,72^. The wind system over India and adjoining oceanic regions generally blows from the southwest for half of the year and from the northeast during the other half; in similar lines, the rainfall pattern and its reversal process are oriented according to the southwest and northeast monsoon seasons^73^. These two Indian monsoon seasons should have a high bearing on various characteristics of the Gulf of Mannar and the Palk Strait, including their patterns of tides and currents, salinity, and chlorophyll distribution^7,27,74,75^. Adam’s Bridge, being a barrier between these two water systems, will be influenced by the transgression of the material energy^76^, resulting in maintaining its current morphological structure. Generally, the West India Coastal Current (WICC) and the Summer Monsoon Current (SMC) bring Arabian Sea waters into the Bay of Bengal during the southwest monsoon, and on the other hand, the East India Coastal Current and Winter Monsoon Current (WMC) carry the Bay of Bengal waters, partly, en route Palk Strait and Adam’s Bridge, into the Arabian Sea during the northeast monsoon^77^.

**Figure 5:**
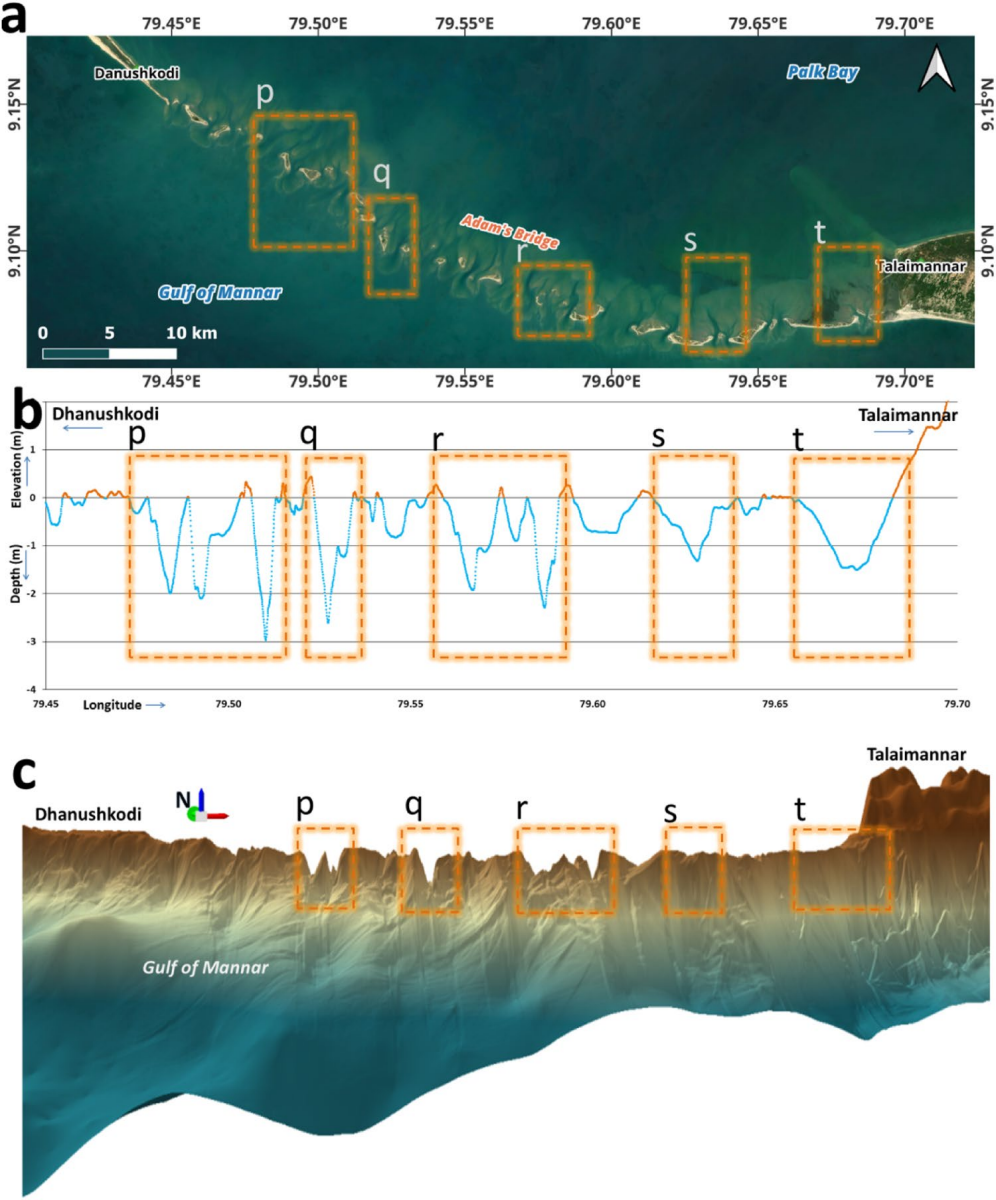
Depiction of the narrow channels along Adam’s Bridge’s crest line. (**a**) Satellite image showing the exposed sand banks along the Adam’s Bridge crest line. (**b**) The Elevation profile along Adam’s Bridge’s crest line shows narrow channels up to a depth of 3 m. (**c**) The three-dimensional perspective of Adam’s Bridge shows the narrow channels enabling water exchange between the Gulf of Mannar and the Palk Strait. *Source* Map in (**a**) is composed in QGIS Ver. 3.30.0-'s-Hertogenbosch (https://qgis.org) using the satellite imagery through web mapping services ()* of the Sentinel-2 cloudless layer for 2021 by EOX ( and ). The perspective view in (c) was generated using ESRI’s ArcScene Ver. 10.8.1 software (). *This work is licensed under a Creative Commons by Attribution (CC BY-NC-SA 4.0) license.

At regular intervals of Adam’s Bridge, sudden narrow channels with depths varying between 2 and 3 m exist (refer to Fig. 5); these narrow channels probably permit free flow or exchange of water between the Gulf of Mannar and the Palk Strait. Importantly, from the crest line of Adam’s Bridge, the narrow channels are accompanied by perpendicular ridges, especially stretching towards the side of Palk Strait; probably, these perpendicular ridges are the result of accumulated sediments/sands that are pushed by the dominant energy waves from the Gulf of Mannar over the years.

Volumetric analysis by fixing 8 m water depth as a base for the study area yielded a volume of ~ 1 km^3^. Out of the total volume of the entire Adam’s Bridge, the volume ratio towards the Gulf of Mannar and Palk Strait is 44:56. Similarly, the volume of Adam’s Bridge above 0 m is 0.02 km^3^ which is only 0.02 percent of the total volume; this is the same extent that is visible in the optical satellite imagery.

## Conclusions

This article reports the physical features of the Adam’s Bridge, an isthmus mostly submerged in shallow and super-shallow waters. Approximately 0.2 million photons representing seafloor depths were aggregated from various qualified ICESat-2 ATL03 data product acquisitions for the Adam’s Bridge region. Additionally, sounding depths from ENCs/charts and elevation data from the open-access bare earth model have contributed to densifying the point database. A 10 m DBEM was generated by interpolating this point database. This DBEM was used in visual analysis aided by 3D perspective views with multi-directional lighting effects. Similarly, the contour and slope map derived from this DBEM and volumetric analysis have aided in quantifying the submerged features of Adams’ Bridge. The report is the first to provide intricate details about the Adam’s Bridge using ICESat-2 water-penetrated photons. Our findings can aid in accentuating the understanding of Adam’s Bridge and its origin.

## Materials and methods

### Datasets

**Figure 6:**
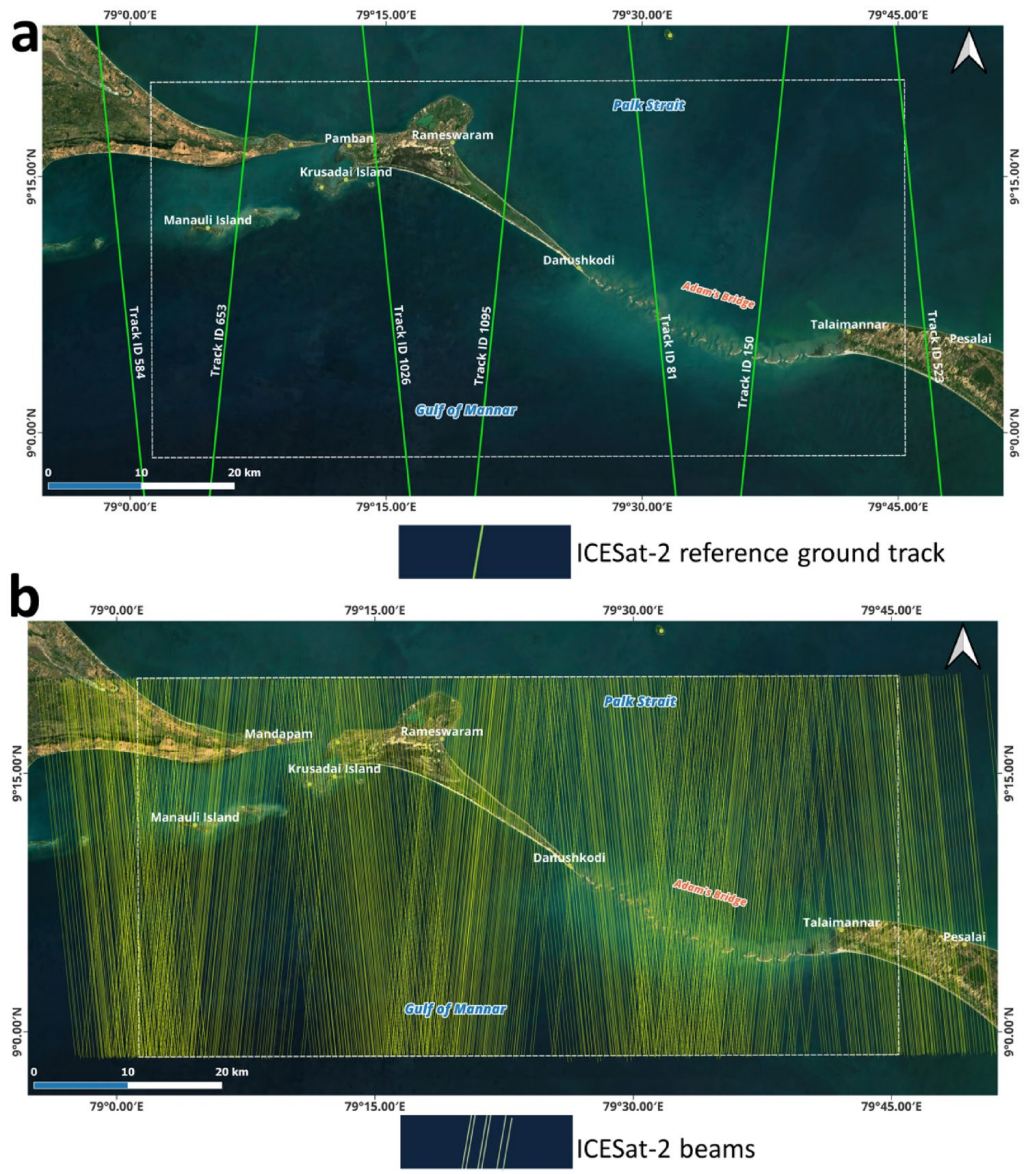
ICESat-2’s reference ground tracks and available beams over the extent of Adam’s Bridge. (**a**) Seven reference ground tracks of ICESat-2 are available over the extent of Adam’s Bridge. (**b**) Available beams (both strong and weak) over the extent of Adam’s Bridge. *Source* Maps are composed in QGIS Ver. 3.30.0-'s-Hertogenbosch (https://qgis.org) using the satellite imagery through web mapping services ()* of the Sentinel-2 cloudless layer for 2021 by EOX ( and ). *This work is licensed under a Creative Commons by Attribution (CC BY-NC-SA 4.0) license.

Table 1 shows the datasets utilized to generate a DBEM for the extent of Adam’s Bridge. The primary data resource to generate the DBEM is depth information of the seafloor retrieved using water-penetrated ICESat-2 photons. Over the extent of Adam’s Bridge, seven ICESat-2 reference ground tracks are available (listed in Table 1 and illustrated in Fig. 6a). From these seven reference ground tracks, one hundred thirty-three ICESat-2 data acquisitions between October 2018 and July 2023 are available for the study area. Out of these, abiding by prerequisite conditions like preferring nighttime acquisitions and omitting the data acquired during turbid load periods (refer to Table 1), 66 tracks are found useful, and these tracks comprise 396 strong and weak beams of along-track data that were considered for this research (shown in Fig. 6b). During ICESat-2 data acquisition process over the water bodies, most photons will be reflected from the surface water. However, depending on the optical properties of the water, some of the photons will return from the water column and some from the seafloor^41,43,48,49,78,79^. Towards retrieving the depth values from the seafloor, preference should be given to those acquisitions of ICESat-2 during clear water (low turbidity) periods^41,54^. For inferring the turbidity load, a data layer titled K_d_(490), available from the Sentinel-3 A/B Ocean and Land Colour Instrument (OLCI) as a Level-2 series of data product service helps to characterize the transparency of water^80–82^. Acquisitions during high turbid load in the water were avoided by referring to the K_d_(490) of Sentinel-3 A/B; during this crosschecking procedure, the overlap period between ICESat-2 and Sentinel-3 A/B acquisitions is kept within + / − 24 h. Similarly, for water depth-related studies using ICESat-2, it is recommended to prefer nighttime acquisitions because the background noise caused by solar spectral radiation significantly impacts the depth detection performance of LiDAR^83,84^.

**Table 1:** Details of the data sources used to generate a digital bathymetric elevation model for the Adam’s Bridge.

Dataset title	Remarks
ICESat-2 Level-2A ATL03 (Geolocated photons)	Source of ICESat-2 ATL03: https://nsidc.org/data/icesat-2
	Ground tracks: 584, 653, 1026, 1095, 81, 150, and 523
	Dates of acquisitions: 1 October 2018 to 30 July 2023
	Preferred seasons of acquisitions: January to May
	Preferred duration of acquisitions: Nighttime
	Preferred beams: Higher preference for the strong beams but cannot omit the photons in the weak beams when there is a need to accumulate denser points to generate a bathymetric surface
	Post-processing methods: Refraction correction applied to the photons returning from the seafloor
	Total number of accrued depths from seafloor returned photons: 0.2 million
K_d_(490) layer from Sentinel-3 A/B mission	Source: https://sentinels.copernicus.eu
	Usage: For the dates acquired by the ICESat-2, within + / − 24 h, corresponding Level-2 Ocean and Land Colour Instrument (OLCI) data products from the Sentinel-3 A/B mission were used to retrieve K_d_(490). K_d_(490) data was used to assess the turbid load in the study area. Only those acquisitions of ICESat-2 were considered while K_d_(490) < 0.12 m^−1^, i.e., clear water conditions
Sounding depths from Electronic Navigational Charts (ENCs) and charts	Source information of ENCs: https://hydrobharat.gov.in, http://www.nara.ac.lk, and https://iho.int
	Usage: The sounding depth values were used to increase the density of depth values in the study area
	Procedure adopted: Sounding depths were digitized from ENCs and chart datasets issued by hydrographic offices
Forest And Buildings removed Copernicus DEM (FABDEM)	Source: https://data.bris.ac.uk
	Usage: Elevation values from FABDEM were used to increase the density of points in the study area for the extent of the land part (Rameshwaram and Talaimannar Islands)
Bathymetric contours (isobaths) generated by Marine Wing, Geological Society of India	Available for an extent at the southern part of Dhanushkodi Island
	Source: Vaz et al.^85^
	Usage: Used as reference data at 14 locations to assess the accuracy of the seafloor depths represented by the ICESat-2 water-penetrated photon
4 m warning contour by i-Boating: Marine Navigation chart	Available near the extent containing the junction of Adam’s Bridge and Talaimannar Island
	Source: https://fishing-app.gpsnauticalcharts.com/
	Usage: Used as reference data at 11 locations to assess the accuracy of the seafloor depths represented by the ICESat-2 water-penetrated photon
Sounding depths from chart number 3040. (Surveyed by Indian Naval Hydrographic Office)	Available at an extent containing the junction of Dhanushkodi Island and Adam’s Bridge
	Source: https://hydrobharat.gov.in/products/online-catalogue/
	Usage: Used as reference data at 10 locations to assess the accuracy of DBEM generated in this research

Additionally, to increase the density and the distribution of the points representing the seafloor’s depth in the regions with less ICESat-2 photons (also, due to the along-track pattern of ICESat-2’s data collection), sounding depths available from ENCs and charts were considered and their details are given in Table 1. Similarly, to increase the point density over the extent of land (Rameshwaram and Talaimannar islands), elevation values were considered from the open-access bare-earth model called FABDEM^58,59^. Various reference datasets were used in the accuracy assessment of the depths retrieved from the ICESat-2 water-penetrated photons and the output DBEM; the details of these reference datasets are given in Table 1.

## Methods

**Figure 7:**
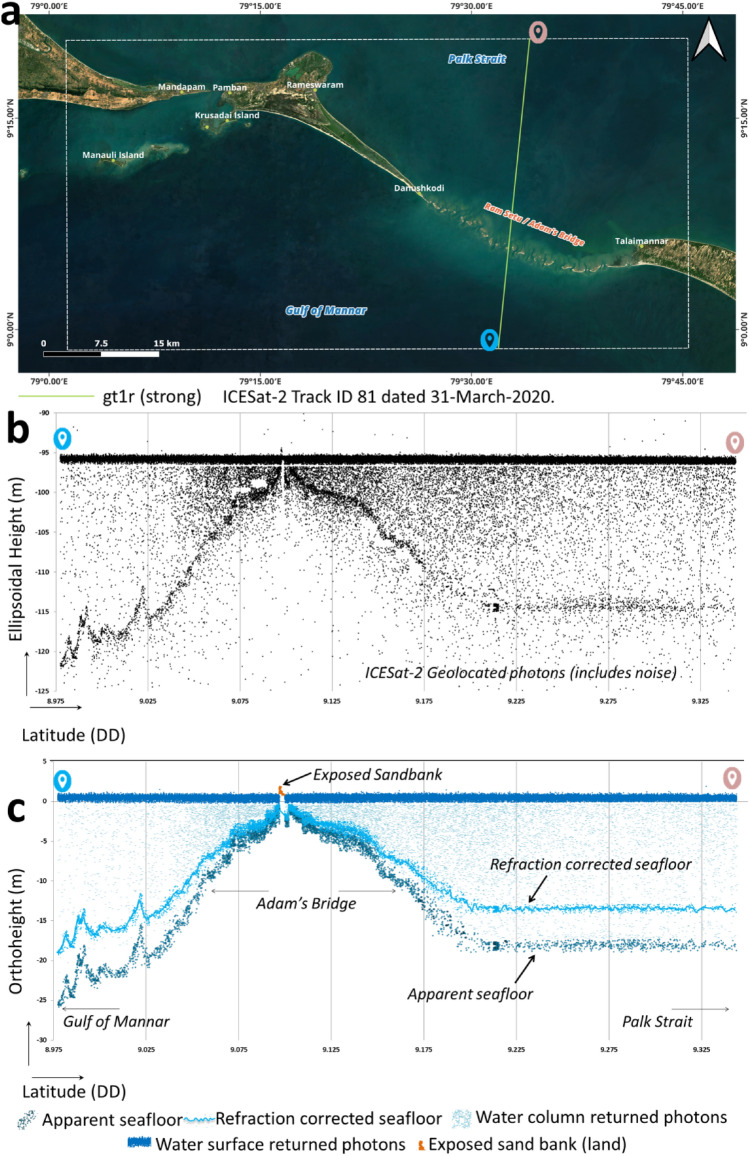
Detection of seafloor from ICESat-2 water-penetrated photons. (**a**) A subset of the ICESat-2 beam acquired over the extent of Adam's Bridge. (**b**) The plot shows the geolocated photons, including noise, that are recorded in the Level-2A ATL03 data product. (**c**) Classification of ICESat-2 photons based on the DBSCAN algorithm; the result consists of photons returned from land, water surface, water column, and seaflooor. The ray tracing mechanism of ICESat-2 photons in the water column will be impacted due to the difference in air and water refraction index as it causes apparent depths; the refraction correction procedure significantly improves the depth estimation accuracy. *Source* Map in (**a**) is composed in QGIS Ver. 3.30.0-'s-Hertogenbosch (https://qgis.org) using the satellite imagery through web mapping services ()* of the Sentinel-2 cloudless layer for 2021 by EOX ( and ). *This work is licensed under a Creative Commons by Attribution (CC BY-NC-SA 4.0) license.

Operational methodologies to derive the seafloor depths from ICESat-2 photons are available from the earlier researchers' works^29,35,41,47–49,52,86–88^, which we have referred to and implemented in this research. We have considered only those ICESat-2 beams that have abided by the prerequisite conditions, like preferring nighttime acquisitions and omitting the data acquired during turbid load periods. All the qualified beams of data were processed to classify the return photons from the water surface, water column, land, and seafloor using Density-Based Spatial Clustering of Applications with Noise (DBSCAN)^84,89–91^ followed by manual correction using localized statistical algorithms^29,91,92^ to eliminate outliers. Typically, the DBSCAN algorithm, by taking parameters like radius and minimum points, will classify a set of points within the said radius as signals when the density of points exceeds the pre-set threshold of minimum points^29^. The concept of retrieving the seafloor returned photons is illustrated in Fig. 7. Figure 7a shows a subset of the ICESat-2 beam acquired over the extent of Adam's Bridge, and Fig. 7b shows its corresponding 2D profile consisting of all the geolocated photons (including noise) as recorded in the ICESat-2 Level-2A ATL03 data product. Typically, these 2D profiles have an x-axis as latitude and a y-axis containing the elevation attributed by the geolocated photons. Figure 7c shows the result of the DBSCAN algorithm, which successfully distinguished the photons returned from land, water surface, water column, and seafloor. By default, photons that have returned from the seafloor are apparent and need applying refraction correction to retrieve their actual depths^41^; this is because there will be a change in the speed of light that occurs at the air–water interface due to the fact that the refractive index of air and seawater is different. In our research, we have implemented the refraction correction to the seafloor returned photons using the method suggested by Dandabathula et al.^29^, Parrish et al.^41^, and Guo et al.^48^; the formula used to perform the refraction correction for the photons that returned from the water column and the seafloor is shown in Eq. (1).

1 $$Dept{h}_{C}= Dept{h}_{apparent}\left.\left( \frac{{n}_{1}}{{n}_{2}}\right.\right)$$

where Depth_C_ is the refraction-corrected depth of the seafloor, Depth_apparent_ is the apparent/default depth (without refraction correction) obtained by computing the difference between the elevation of the water surface (moving average of the surface waves) and the seafloor, and n_1_ ≈ 1.00029 and n_2_ ≈ 1.34116 are the refractive index of air and water bodies. The refracted corrected photons returned from the seafloor are shown in Fig. 7c. Photons returned from the water surface and water column were discarded during the computation of bathymetry as they do not have any role in estimating the seafloor depth.

**Figure 8:**
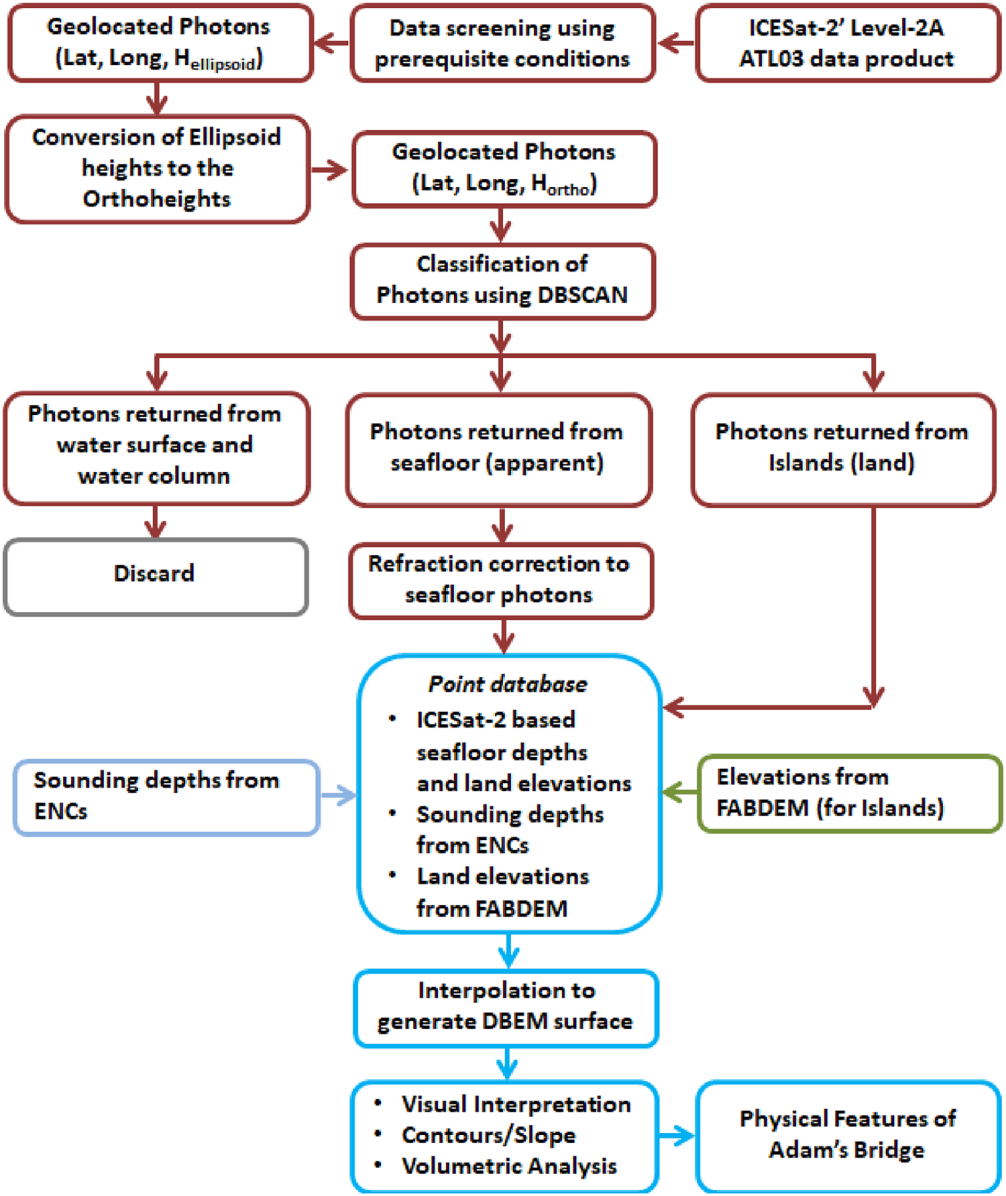
Schematics diagram of methodology to generate a digital bathymetric elevation model from ICESat-2 photons.

The default vertical datum of ICESat-2 ATL03, i.e., heights above the WGS84 ellipsoid, was converted to orthometric heights by preferring the EGM2008 geoid model in the geoid height calculator available at https://www.unavco.org. By extending this method to all the 396 beams over the study area, approximately 0.2 million ICESat-2 photons representing the seafloor’s depth values and terrain elevations were collected as a part of data collection from the ICESat-2 ATL03 data products. Figure 8 shows the schematic representation of the methodology implemented in this research towards generating a DBEM for Adam’s Bridge, which is partly modified from earlier researchers’ works^29,35,41,47–49,52,86–88^.

**Figure 9:**
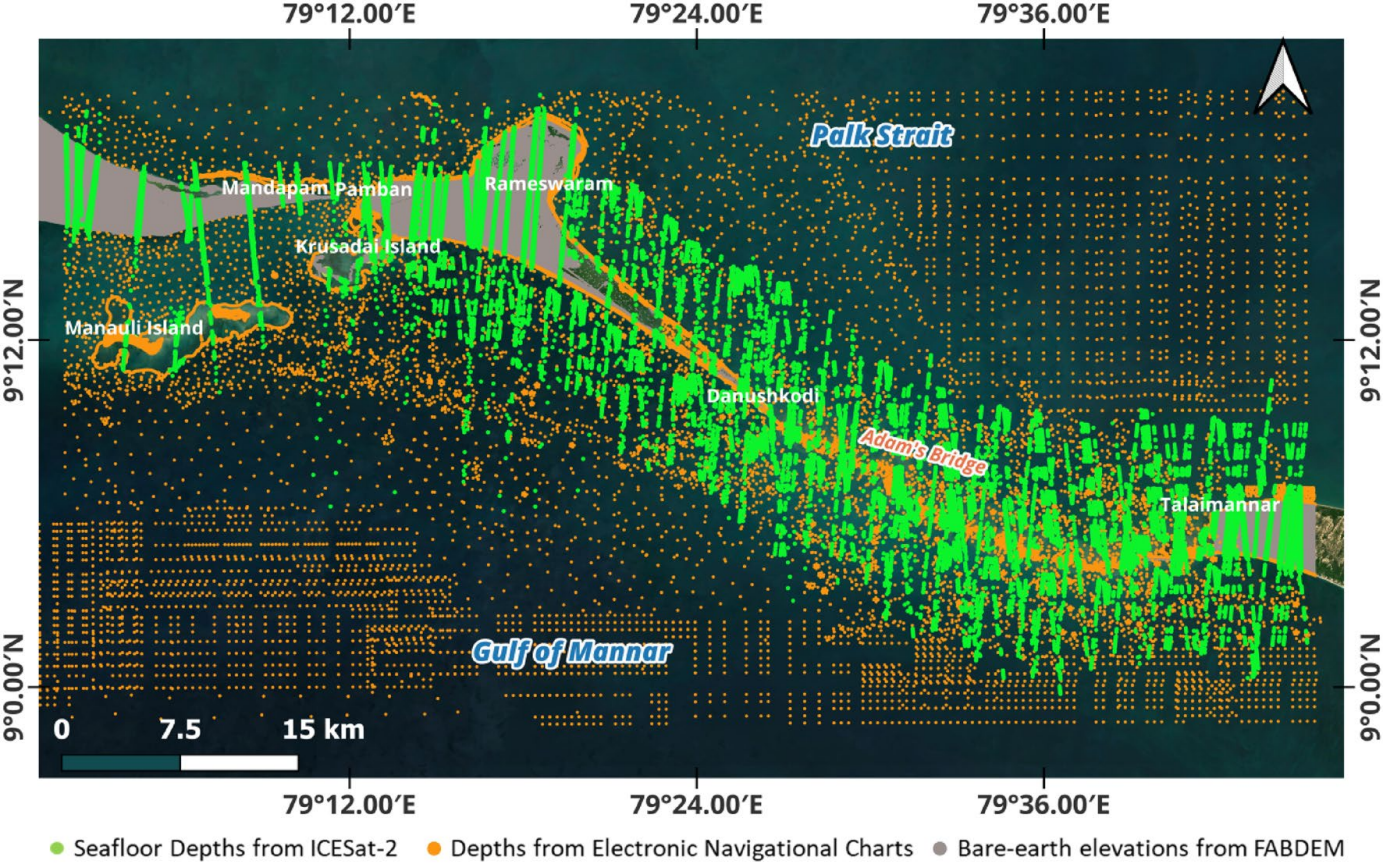
Distribution of points representing the depths accrued from seafloor returned, and refraction corrected ICESat-2 photons, sounding depths from ENCs/charts, and land elevation values from FABDEM.

Interpolation is essential in generating bathymetric surfaces from known depth points; the process enables estimating the depths in areas lacking direct measurements. The point database containing the accumulated depths and elevation values from the ICESat-2 photons must undergo an interpolation procedure to generate a continuous surface. Due to the along-track pattern of data collection by ICESat-2, interpolation techniques may not yield a continuous surface; for this, additional points representing seafloor depths are needed in the regions with gap areas. The additional depths were considered from available ENCs and charts (mentioned in Table 1). Similarly, to increase the point density over the extent of land (Rameshwaram and Talaimannar islands), additional elevation values (converted to orthoheights) were considered from FABDEM^58^. Figure 9 shows the distribution of seafloor depths accumulated from the processed ICESat-2 photons and ENCs/charts, along with the elevation values for Islands from FABDEM. A few points from the ENCs/charts were reserved for technical validation (considered as checkpoints) of the output DBEM (discussed in the subsequent section) and not included as a part of the above-said point database.

### Validation of depths derived from ICESat-2 water penetrated photons

**Figure 10:**
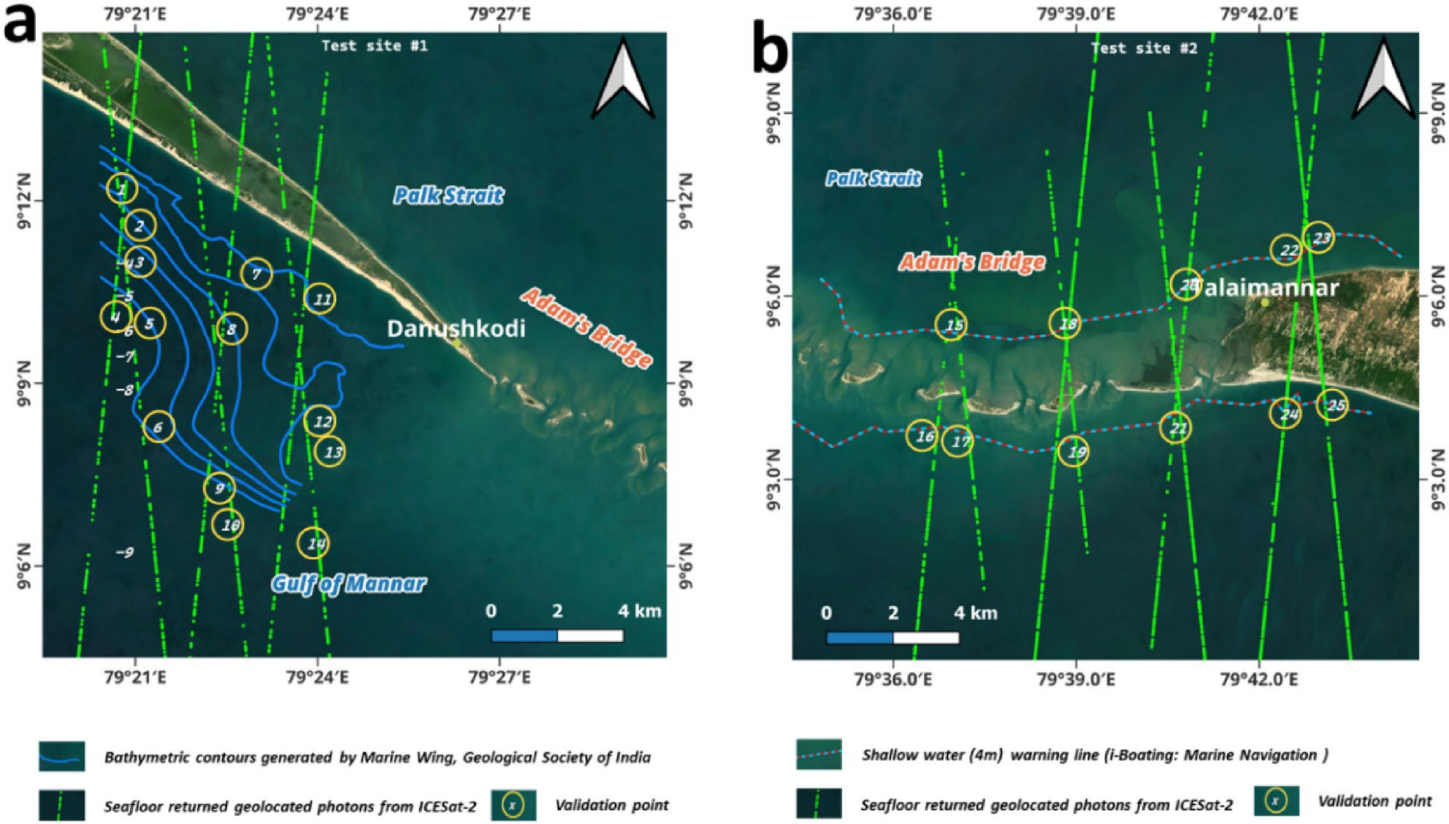
Test sites to evaluate the bathymetric performance of ICESat-2 geolocated photons. (**a**) Test site 1 shows the extent of southern Dhanushkodi with GSI-surveyed bathymetric contours overlaid with the ICESat-2 beams. (**b**) Test site 2 shows the extent of Adam’s Bridge and Talaimannar with i-Boating: Marine Navigation-based 4 m shallow water warning line overlaid with the ICESat-2 beams. *Source* Maps are composed in QGIS Ver. 3.30.0-'s-Hertogenbosch (https://qgis.org) using the satellite imagery through web mapping services ()* of the Sentinel-2 cloudless layer for 2021 by EOX ( and ). *This work is licensed under a Creative Commons by Attribution (CC BY-NC-SA 4.0) license.

Towards assessing the accuracy of the depths obtained from the ICESat-2 water-penetrated photons, technical validation has been performed at two test sites that are falling in the study area of this research (shown in Fig. 10). High accurate reference data is needed to compare the depths obtained from ICESat-2 photons. However, due to the shallow nature of the water around the Adam’s Bridge, no research vessel could attempt to negotiate the dangers associated with the area. To date, geological studies around the Adam’s Bridge are meager except for a limited geomorphological observation made by the Marine Wing of the Geological Survey of India (GSI) at the southern part of Dhanushkodi Island^85^ and a seismic survey by the National Institute of Oceanography, Council of Scientific and Industrial Research at the northern extent of Dhanuskhodi Island^68^. In particular, GSI, as a part of the geomorphological observation, conducted a detailed bathymetric survey for an area of about 12 × 2 km by engaging a mechanized wooden boat fitted with a portable echo sounder^85^. Bathymetric contours resulting from this survey were used as reference data for test site 1 (refer to Fig. 10a). Similarly, i-Boating: Marine Navigation offers marine charts through its application that offers offline nautical charts, inland river navigation charts, and lake contour maps for fishing, kayaking, yachting, and sailing (available as a subscription service at https://fishing-app.gpsnauticalcharts.com/). The navigation chart offers a warning contour at 4 m depth in shallow water near the extent of Adam’s Bridge and Talaimannar (refer to Fig. 10b); this warning contour at 4 m depth prompts large draught boats not to navigate beyond the safety contour. This warning contour at a depth of 4 m is used as reference data in test site 2. A total of 14 ICESat-2 beams fell in the extent of these two test sites comprising the reference data sets. The ICESat-2 photons from these 14 beams were overlapping at 25 locations. The values of seafloor depths from ICESat-2 and contour values of reference data were compared to validate the accuracy (shown in Fig. 10 and listed in Table 2) at these 25 locations using Root Mean Square Error (RMSE), a statistical quantifier of error. Table 2 shows the depth values derived from the ICESat-2 water-penetrated photons and their differences with respect to the reference data. Root Mean Square Error (RMSE) shows how far predictions fall from true values using Euclidean distance. The formula used to compute RMSE is shown in Eq. (3).

2 $$\Delta H={(\text{Depth})}_{ICESat-2}- {(\text{Depth})}_{\text{reference}}$$

3 $$RMSE= \sqrt{\frac{\sum {\Delta H}^{2}}{n}}$$

where (Depth)_ICESat-2_ is the set of depth values obtained from the ICESat-2 water-penetrated photons and (Depth)_reference_ is the set of depths considered from the reference datasets. n (= 25) is the number of observations.

**Table 2:** Values of depths from water penetrated ICESat-2 photons and reference datasets.

Validation point ID	Test site details	Details of ICESat-2 beam (Track ID, Beam ID & date of acquisition)	Location of the photon (Lat, Long)	Depth from water penetrated ICESat-2 photon (m)	Depth from the reference contour (m)	Difference (m)
1	Test site #1	1095, gt1l (strong) & 2019-03-10	8.978989293, 79.92175472	− 5.29	− 5	0.29
2		81, gt1r (strong) & 2021-09-27	9.251366434, 79.46589798	− 5.76	− 6	− 0.24
3		81, gt1r (strong) & 2021-09-27	9.245215592, 79.46527704	− 6.95	− 7	− 0.05
4		81, gt1r (strong) & 2021-09-27	9.228075287, 79.46355104	− 9.17	− 9	0.17
5		1095, gt1l (strong) & 2019-03-10	8.945058751, 79.92517938	− 9.11	− 9	0.11
6		1095, gt1l (strong) & 2019-03-10	8.909116639, 79.92879926	− 9.27	− 9	0.27
7		81, gt3l (strong) & 2019-06-30	9.211753502, 79.46189763	− 3.94	− 4	− 0.06
8		1095, gt2l (strong), 2019-06-08	9.191901847, 79.45989843	− 5.72	− 6	− 0.28
9		81, gt3l (strong) & 2019-06-30	9.150750419, 79.45574654	− 8.09	− 8	0.09
10		81, gt3l (strong) & 2019-06-30	9.147057327, 79.45537558	− 9.18	− 9	0.18
11		Track ID: 81, gt3l (strong) & 2019-04-03	9.27153036, 79.46792697	− 4.27	− 4	0.27
12		81, gt3l (strong) & 2019-04-03	9.240765576, 79.46482647	− 5.29	− 5	0.29
13		1095, gt1l (strong) & 2019-06-08	8.881697857, 79.93155789	− 4.84	− 5	− 0.16
14		81, gt3l (strong) & 2019-04-03	9.209845333, 79.46171314	− 8.21	− 8	0.21
15	Test site #2	150, gt3r (strong) & 2020-10-03	9.088207745, 79.61792814	− 3.76	− 4	− 0.24
16		523, gt3r (strong) & 2020-01-29	9.06172263, 79.61182362	− 4.08		0.08
17		150, gt3r (strong) & 2020-10-03	9.060228565, 79.62075552	− 3.82		− 0.18
18		150, gt2r (strong) & 2020-10-03	9.091009336, 79.64777263	− 4.18		0.18
19		523, gt2r (strong) & 2020-01-29	9.056401869, 79.64428682	− 3.92		− 0.08
20		523, gt1r (strong) & 2020-01-29	9.099878329, 79.68016254	− 3.96		− 0.04
21		150, gt1r (strong) & 2020-10-03	9.06462907, 79.67871202	− 4.28		0.28
22		523, gt3l (strong) & 2019-01-31	9.112412382, 79.71154952	− 4.24		0.24
23		150, gt3l (strong) & 2022-04-01	9.112766351, 79.71374143	− 4.28		0.28
24		523, gt3l (strong) & 201-01-31	9.066738176, 79.70693504	− 3.85		− 0.15
25		150, gt3l (strong) & 2022-04-01	9.069453917, 79.71811012	− 4.18		0.18
Root Mean Square Error (RMSE) (m)						0.2

RMSE resulting from these 25 validation points is 0.2 m. Earlier, researchers performed the accuracy assessment of the seafloor depths derived from the ICESat-2 photons for shallow water, and it is proven that the performance is as per the sensor specifications^52^, and generally, during clear water conditions, the accuracy in terms of RMSE is in the range of 0.2–0.8 m^41,45,49^. These accuracies can be termed significantly high accurate measurements from the currently available operational space-borne active sensor systems. Moreover, the accuracy assessment from our research, which yielded an RMSE of 0.2 m, is also in the similar lines of assessment done by earlier researchers, indicating significant potential for bathymetric mapping with ICESat-2 photons, especially for the shallow waters.

### Generation of DBEM and its accuracy assessment

The point database containing depths and the elevations of the study area were interpolated using the Inverse Distance Weighted (IDW) method; this method is considered a highly adaptable estimation method as it is best to reconstruct natural surface recourses given dense and well-distributed points in the study area^93,94^. IDW interpolation was done using a Geographic Information System software, ESRI’s ArcGIS Pro 10.3 version (http://www.esri.com). The resolution of a bathymetric elevation model refers to the cell/grid size of the raster; smaller grids represent higher resolution and more detail. Determination of the cell size of a DEM is a fundamental component during a surface generation process as it aims to produce the best representation of a terrain surface without introducing artifacts from interpolation. A standard method for determining the cell size of a raster-based elevation model has been defined by Hu^95^ and endorsed by Langridge et al.^96^ is shown in Eq. (4)

4 $$s=\sqrt{A/n}$$

where s is the estimated raster cell size, and n is the number of points in the minimum area of density (A) within the extent of the point distribution. From the point distribution map obtained in our experiment, it is observed that for every 200 sq.m, at least 2 points representing depth/elevation exist. Thus, the output pixel's cell size was set to 10 m during the interpolation stage based on Eq. (4).

**Figure 11:**
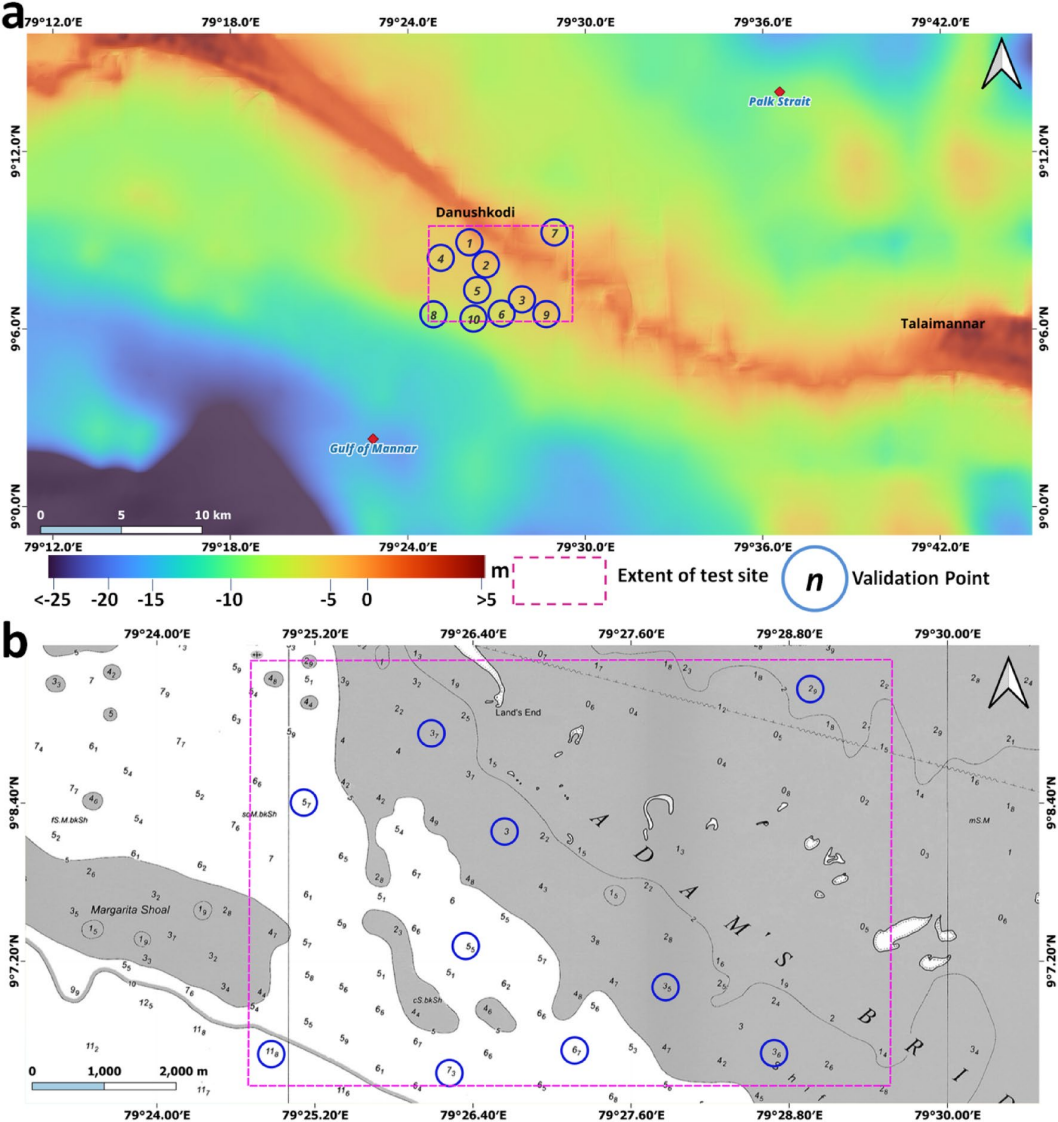
Accuracy assessment of digital bathymetric elevation model (DBEM) generated with collection of depths accrued from ICESat-2 geolocated photons and ENCs. (**a**) DBEM showing the test site (marked in magenta) containing locations of validation points (**b**) Chart showing sounding depths and locations of validation points.

By applying the processing scheme as mentioned above, we generated the high-resolution DBEM for the Adam's Bridge, in which ~ 0.2 million points are from the ICESat-2 photons have played a significant role in the surface generation along with the additional points from ENCs/charts and FABDEM (elevation points for Islands). During this process, a few values of sounding depths (considered from INHO chart number 3040) were reserved as checkpoints towards quality checking of the output DBEM, which were not used in the interpolation. The distribution of these checkpoints is shown in Fig. 11. RMSE, a statistical formula, was used to quantify the accuracy of seafloor depth. Table 3 shows the depths retrieved from the DBEM generated in this research (shown in Fig. 11a) along with the sounding depths considered from the reference chart (shown in Fig. 11b). RMSE computed from these validation points resulted in a value of 0.25 m (or 0.82 ft.) with a minimum and maximum difference obtained from the individual validation points as 0.1 m and 0.59 m respectively. The result suggests that ICESat-2-based bathymetric data accuracy may be sufficient for studying the benthic features in shallow waters with accuracy specification within a foot and where ship-borne bathymetric data might otherwise be applied.

**Table 3:** Comparison of depth values retrieved from Indian National Hydrographic Office chart and ICESat-2 photons based digital bathymetric elevation model.

Validation point ID	Location of the photon (Lat, Long)	Depth from DBEM (m)	Sounding Depth from the chart (m)	Difference (m)
1	9.148812846, 79.434725590	− 3.54	− 3.7	− 0.16
2	9.136398738, 79.443993265	− 3.10	− 3	0.10
3	9.116719231, 79.464359267	− 3.39	− 3.5	− 0.11
4	9.140060042, 79.418592971	− 5.88	− 5.7	0.18
5	9.121925147, 79.439073388	− 5.22	− 5.5	− 0.28
6	9.108710129, 79.452860485	− 6.11	− 6.7	− 0.59
7	9.154419217, 79.482665786	− 3.02	− 2.9	0.12
8	9.108252466, 79.414474004	− 11.56	− 11.8	− 0.24
9	9.108366881, 79.478089156	− 3.49	− 3.6	− 0.11
10	9.105849735, 79.437013905	− 7.08	− 7.3	− 0.22
Root Mean Square Error (RMSE) (m)				0.25

The resulting DBEM was used for visual inspection through 3D perspective views with multi-directional lighting effects, which has helped enhance the impressions of relief; for this, the visualization platform available in ESRI’s ArcScene software (www.esri.com) was used for interpretation. The same DBEM has been used to derive contour and slope maps and to perform volumetric analysis.

## Data Availability

Level-2A ATL03 data product of ICESat-2 can be found in the NASA National Snow and Ice Data Center Distributed Active Archive Centre (NSIDC DAAC) available at https://nsidc.org/data/icesat-2. Data products associated with Sentinel-3 A/b can be downloaded from https://sentinels.copernicus.eu/web/sentinel/missions/sentinel-3/data-products/olci maintained by Copernicus Data Space Ecosystem, European Space Agency. Hydrographic data associated with the study area are available at https://hydrobharat.gov.in/online-catalogue-application/ and can be procured through authorised chart agents listed at https://hydrobharat.gov.in/products/chart-agents/. The Digital Bathymetric Elevation Model (DBEM) described in this study is available from the corresponding author upon reasonable request.
